# Empagliflozin on top of metformin treatment improves arterial function in patients with type 1 diabetes mellitus

**DOI:** 10.1186/s12933-018-0797-6

**Published:** 2018-12-03

**Authors:** Mojca Lunder, Miodrag Janić, Miha Japelj, Andrej Juretič, Andrej Janež, Mišo Šabovič

**Affiliations:** 1 Department of Endocrinology, Diabetes and Metabolic Diseases, University Medical Centre Ljubljana, Zaloška Cesta 7, 1000 Ljubljana, Slovenia; 2 Department of Vascular Diseases, University Medical Centre Ljubljana, Zaloška Cesta 7, 1000 Ljubljana, Slovenia

**Keywords:** Empagliflozin, Metformin, Empagliflozin/metformin, Vascular protection, Arterial stiffness, Endothelial function

## Abstract

**Background:**

Deteriorated arterial function and high incidence of cardiovascular events characterise diabetes mellitus. Metformin and recent antidiabetic drugs, SGLT2 inhibitors, reduce cardiovascular events. We explored the possible effects of empagliflozin’s effect on top of metformin treatment on endothelial function and arterial stiffness parameters in type 1 diabetes mellitus (T1DM) patients.

**Methods:**

Forty T1DM patients were randomised into three treatment groups: (1) empagliflozin (25 mg daily), (2) metformin (2000 mg daily) and (3) empagliflozin/metformin (25 mg daily and 2000 mg daily, respectively). The fourth group received placebo. Arterial function was assessed at inclusion and after 12 weeks treatment by: endothelial function [brachial artery flow-mediated dilation (FMD), reactive hyperaemia index (RHI)], arterial stiffness [pulse wave velocity (PWV) and common carotid artery stiffness (β-stiffness)]. For statistical analysis one-way analysis of variance with Bonferroni post-test was used.

**Results:**

Empagliflozin on top of metformin treatment significantly improved endothelial function as did metformin after 12 weeks of treatment: FMD [2.6-fold (P < 0.001) vs. 1.8-fold (P < 0.05)] and RHI [1.4-fold (P < 0.01) vs. 1.3-fold (P < 0.05)]. Empagliflozin on top of metformin treatment was superior to metformin in improving arterial stiffness parameters; it significantly improved PWV and β-stiffness compared to metformin [by 15.8% (P < 0.01) and by 36.6% (P < 0.05), respectively]. Metformin alone did not influence arterial stiffness.

**Conclusion:**

Empagliflozin on top of metformin treatment significantly improved arterial stiffness compared to metformin in T1DM patients. Endothelial function was similarly improved in all treatment groups. Empagliflozin seems to possess a specific capacity to decrease arterial stiffness, which could support its cardioprotective effects observed in large clinical studies.

*Trial registration* Clinical trial registration: NCT03639545

## Background

Diabetes mellitus is a systemic, chronic metabolic disease that affects all organs of the body. Of particular importance is the effect of diabetes on the cardiovascular system, where it makes the arteries prone to microvascular as well as macrovascular complications. It is well-known that cardiovascular diseases represent the most common factor for diabetes mellitus-related morbidity and mortality [1]. The mechanisms through which diabetes deteriorates the arterial function are highly studied and mostly understood. Primarily, the functional and structural arterial wall characteristics become impaired, characterised by endothelial dysfunction and increased arterial stiffness. These changes appear through the arterial wall molecules glycosylation, intensive inflammation and oxidative stress. These processes are accelerated in diabetes mellitus patients and appear earlier in the disease course compared to other populations [2]. Both endothelial dysfunction and arterial stiffness are causally involved in cardiovascular events and are good predictors of cardiovascular events [3–5]. Therefore, they are both very prone targets for intervention aiming to decrease cardiovascular events in diabetic patients [6].

In recent years, the treatment goals of diabetes patients have been broadened from merely glycaemia control to cardiovascular protection. The newest drugs in the antihyperglycemic group are sodium glucose co-transporter type 2 (SGLT2) inhibitors, which are currently registered only for treatment of type 2 diabetes mellitus (T2DM) patients. Basically, these drugs increase glucose excretion through the urine and consequently decrease blood glucose values through the inhibition of SGLT2 channels in the kidneys [7]. During cardiovascular safety trials, performed at the request of the Food and Drug Administration (FDA), they were proved to have significant cardioprotective properties. In two large studies, empagliflozin (EMPA-REG study) and canagliflozin (CAVNAS study) reduced major adverse cardiovascular events by 14% in T2DM patients. Importantly, the precise mechanisms of these cardioprotective effects remains not fully understood [8, 9]. On the other hand, the cardiovascular efficacy of metformin was proven in the UKPDS80 trial that showed a significant reduction of myocardial infarctions by 39% in T2DM patients [10].

The beneficial effects of SGLT2 inhibitors on arterial function have been established in prior studies [11–13]. However, no systematic studies exploring all arterial system functions, i.e. from endothelial function to arterial stiffness, have yet been performed. Furthermore, no comparison and/or study of the joint effects with metformin, which is a basic drug for treatment of T2DM patients, has been performed. Such comparison could result in useful data, since these are two seemingly different agents with a cardiovascular protective capacity. A study with concomitant use of both agents could therefore represent a useful model to better understand the mechanism(s) underlying their cardiovascular protective effects, which could speculatively be similar, different or cumulative. The aim of the present study was to compare the treatment effects of empagliflozin on the top of metformin to metformin on endothelial function and arterial stiffness in T1DM patients.

## Methods

### Study design

In this 12-week, prospective, double-blind randomised clinical study forty male T1DM patients from Diabetes outpatient clinic Ljubljana were recruited. All patients were treated with insulin, either through intensive insulin therapy or insulin pump therapy. The patients were equally randomised into three treatment groups, that received the treatment in addition to insulin. The groups were as follows: (1) empagliflozin group (receiving 25 mg daily), (2) metformin group (receiving 2000 mg daily) and (3) empagliflozin/metformin group (receiving empagliflozin 25 mg daily and metformin 2000 mg daily). The fourth group received placebo. The duration of the study period was 12 weeks. All subjects voluntarily participated in this study and signed an informed consent. The study was approved by the National Medical Ethics Committee of Slovenia. The study was registered at http://clinicaltrials.gov (NCT03639545).

### Study population

For inclusion in the study, patients needed to have a confirmed T1DM diagnosis and be between 30 and 65 years of age. Exclusion criteria included diagnosed advanced heart failure (left ventricular ejection fraction below 40%, NYHA class II–III, NT-proBNP values above 500 pg/ml), kidney (estimated glomerular filtration rate below 60 ml/min/1.73 m^2^) or liver failure, benign prostatic hyperplasia, prostatic carcinoma, frequent urinary tract infections or body mass index below 23 kg/m^2^.

### Endpoints

The aim of this study was to explore the effect of empagliflozin on top of metformin treatment on arterial function parameters after 12 weeks of treatment.

### Study protocol

At the beginning of the study, a complete history was taken and full medical examination of each patient was performed. At inclusion in the study and after 12 weeks of treatment, arterial function measurements were performed, comprising (i) measurements of endothelial function [brachial artery flow-mediated dilation (FMD), reactive hyperaemia index (RHI)]; and (ii) measurements of arterial stiffness [carotid artery pulse wave velocity (cPWV), carotid-femoral PWV (cfPWV) and common carotid artery stiffness (β-stiffness)]. Additionally, venous blood samples were obtained at the beginning and at the end of the study. Glycated haemoglobin (HbA1c) was assessed using the VITRO 5.1FS Chemistry System (Ortho Clinical Diagnostics, Raritan, New Jersey). An automated sphygmomanometer was used for blood pressure measurements. Arterial function assessment was performed in standardised conditions, in a quiet and temperature-controlled environment. All the patients were subject to prior 8 h fasting (the measurements were performed in the early morning, before the patients’ first meal). Alcohol and caffeine abstinence were also required. Each of the measurements were performed at the same time and by the same examiner, both at the beginning and the end of study. Ultrasound measurements were obtained using an Aloka ProSound α7 machine with an integrated high resolution eTracking system. Reactive hyperaemia index was measured using an Endopat 2000 device (Itamar Medical Ltd., Caesarea, Israel), while cfPWV was obtained using a SphygmoCor device (AtCor Medical, Sydney, Australia) with SphygmoCor CvMS software (version 9). Continuous electrocardiogram recording was also performed during all measurements.

### Endothelial function assessment

#### Brachial artery flow-mediated dilation (FMD) assessment

Endothelial function was assessed in accordance with current guidelines [14] through brachial artery FMD. Patients laid supine with their right arm extended and fixed on the examination table with rubber foam. Blood pressure was recorded using the other arm. An additional blood pressure cuff was used on the right forearm. The brachial artery was recorded by ultrasound and its diameter was obtained by using the machine’s continuous tracking software. Baseline diameter was obtained for 1 min, and after that, the blood pressure cuff was inflated to 50 mmHg above the systolic blood pressure for 4 min, resulting in the occlusion of the forearm arteries. After that time, the blood pressure cuff was rapidly deflated, inducing reactive hyperaemia. The brachial artery diameter was then continuously recorded for another 3 min. At the end of examination, the ultrasound machine automatically generated the values of baseline and maximal brachial artery diameter and FMD.

#### Reactive hyperaemia index (RHI) assessment

Plethysmography measurement of finger arterial pulse wave amplitude was measured using an Endopat 2000 device [15]. Patients laid in a semi supine position with their forearms extended on a foam arm-rest under a 40º angle as prescribed by the manufacturer. Pneumatic probes were placed on both index fingers and inflated to 70 mmHg. The baseline signal was recorded for 5 min, followed by blood pressure cuff inflation on the left forearm to 60 mmHg above systolic blood pressure for another 5 min. The blood pressure cuff was then rapidly deflated, inducing reactive hyperaemia for the final 5 min. RHI was automatically calculated through the ratios between pulse wave amplitudes during the reactive hyperaemia phase and baseline phase.

### Arterial stiffness assessment

#### Carotid pulse wave velocity (cPWV) and β-stiffness assessment

Both measurements were performed on the right common carotid artery. Patients laid in a supine position, their head additionally elevated by 45º and tilted to the side by 30º. Using the device’s software, stiffness was measured through analysis of the pulse waves obtained. The tracker pair was fixed at the anterior and posterior wall of the common carotid artery. The machine obtained the pressure waveforms from the changing arterial diameters that were calibrated based on systolic and diastolic blood pressure. Both cPWV and β-stiffness were then calculated automatically as a mean of 12 beats.

#### Carotid-femoral pulse wave velocity (cfPWV) assessment

A Sphygmocor with a CvMS software device was used in accordance to the Consensus document on arterial stiffness for measurement of cfPWV [16]. The latter was calculated automatically as the ratio of the pulse wave distance travelled and the transit time needed. The pulse waveforms were obtained from the right common carotid artery and right femoral artery, while the distance was calculated as the difference between the distance from the sternal notch to the common carotid artery and from the sternal notch to the femoral artery, again in accordance with the guidelines.

### Statistical analysis

The values were expressed as mean ± SEM. Their differences were assessed by one-way analysis of variance (ANOVA). For significant interactions, the Bonferroni post-test was performed. A P-value of less than 0.05 was considered statistically significant. Statistical analyses were processed using the GraphPad Prism 5.0 software. Due to a small number of participants, eligible arterial stiffness parameters, i.e. cPWV and cfPWV, were combined into one (PWV), thus increasing the results’ value.

## Results

### Patient characteristics

Patient characteristics are presented in Table 1. The age of the patients, duration of diabetes mellitus and smoking habits did not differ between groups. There were 20% of smokers in each study group. The studied groups did not differ in prior cardiovascular events, which were present in 10% of patients in each group. Other characteristics of the individual study groups are presented as values at the beginning of the study and after the 12-week treatment period. Empagliflozin/metformin significantly decreased HbA1c after 12 weeks of treatment (for 0.6%, P < 0.05) (Table 1). No significant differences in systolic and diastolic blood pressure, body mass index or waist circumference were found between the groups.

**Table 1:** Patient characteristics in study groups at inclusion in the study (before) and after 12 weeks of treatment (after) with empagliflozin 25 mg daily (EMPA), metformin 2000 mg daily (MET), empagliflozin/metformin (EMPA/MET) or placebo (PLACEBO)

	PLACEBO		EMPA		MET		EMPA/MET	
	Before	After	Before	After	Before	After	Before	After
Average age (years)	43.1 ± 2.1		46.0 ± 2.3		46.4 ± 3.9		43.3 ± 2.6	
Duration of diabetes (years)	22.2 ± 3.8		22.5 ± 3.7		23.2 ± 4.8		22.3 ± 3.2	
HbA1c (%)	7.8 ± 0.2	7.7 ± 0.3	7.8 ± 0.1	7.4 ± 0.1	7.9 ± 0.2	7.7 ± 0.3	7.8 ± 0.2	7.2 ± 0.2*
Systolic BP (mmHg)	129.9 ± 4.0	127.4 ± 2.9	134.5 ± 3.0	127.2 ± 2.9	132.4 ± 3.1	125.9 ± 2.1	129.9 ± 2.6	124.8 ± 3.4
Diastolic BP (mmHg)	73.2 ± 5.4	77.0 ± 2.4	83.8 ± 2.6	81.9 ± 2.4	79.1 ± 1.5	79.8 ± 2.3	84.4 ± 1.1	73.7 ± 1.8
BMI (kg/m^2^)	28.3 ± 0.5	28.5 ± 0.31	28.9 ± 0.7	28.4 ± 0.6	28.0 ± 0.3	27.5 ± 0.3	28.9 ± 0.9	27.7 ± 1.0
Waist circumference (cm)	96.9 ± 3.9	95.2 ± 3.5	101.4 ± 2.9	97.1 ± 2.6	97.8 ± 4.0	97.9 ± 4.9	99.1 ± 3.9	97.7 ± 3.6

Values are expressed as mean ± standard error of the means

BP, blood pressure; BMI, body mass index; HbA1c, glycated haemoglobin

* P < 0.05 for comparison within the group

### Effect on endothelial function

**Fig. 1:**
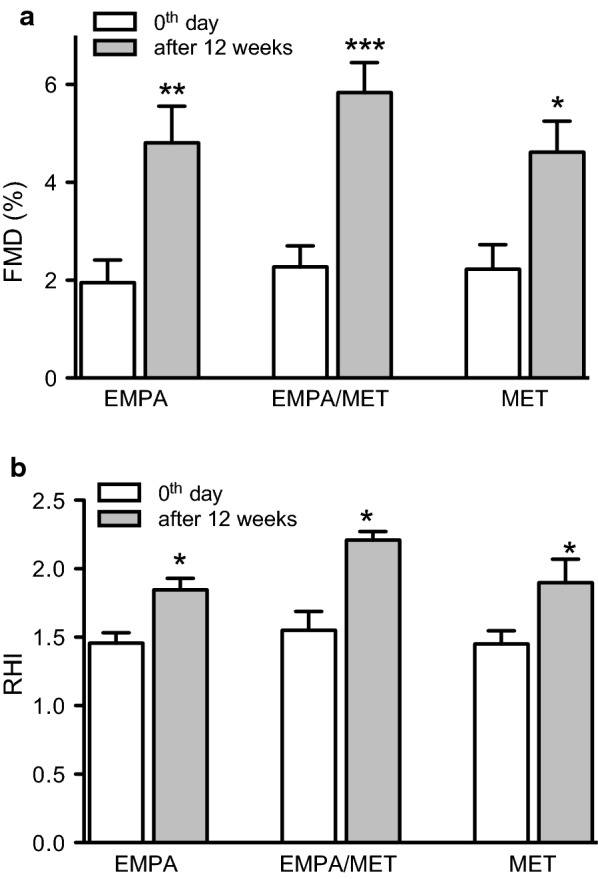
**a** Brachial artery flow-mediated dilation (FMD) values and **b** reactive hyperaemia index (RHI) at inclusion in the study (day 0; white columns) and after 12 weeks of treatment (grey columns) in groups treated with empagliflozin 25 mg daily (EMPA), metformin 2000 mg daily (MET) or empagliflozin/metformin (EMPA/MET). *P < 0.05; **P < 0.01; ***P < 0.001 for comparison with initial values at the beginning of the treatment in separate groups

Brachial artery FMD significantly improved after 12-weeks treatment with empagliflozin (up to 2.2-fold, P < 0.01), metformin (up to 1.8-fold, P < 0.05) and with empagliflozin/metformin (up to 2.6-fold, P < 0.001) compared to basal values in separate groups. On the other hand, there were no significant differences in FMD improvements between the treatment groups (Fig. 1a). In the placebo group, no FMD changes were observed during the course of the study.

Reactive hyperaemia index (RHI) significantly improved with empagliflozin (up to 1.3-fold, P < 0.01), metformin (up to 1.3-fold, P < 0.05) and empagliflozin/metformin combination (up to 1.4-fold, P < 0.01). There were no significant differences in RHI improvements between the treatment groups (Fig. 1b). During the study course, no changes of RHI were observed in the placebo group.

### Effects on arterial stiffness

**Fig. 2:**
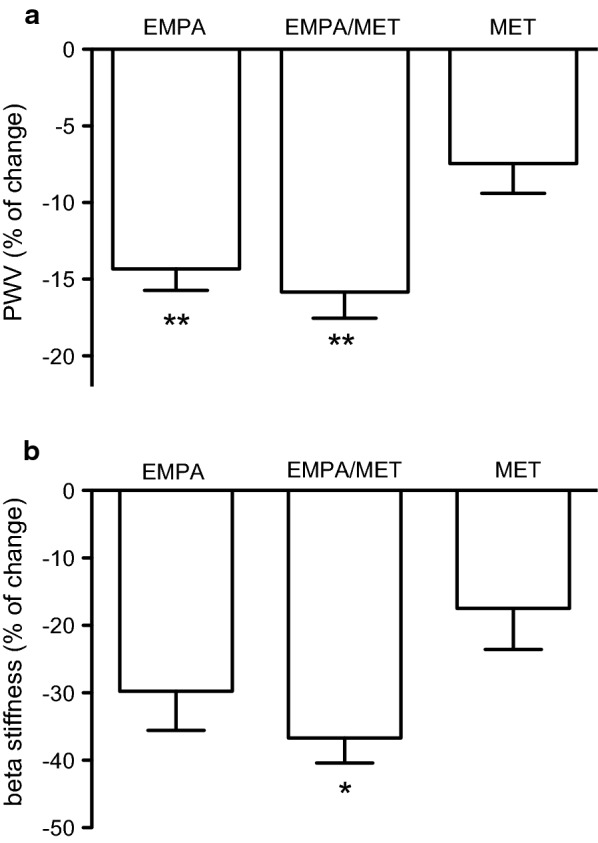
**a** Pulse wave velocity (PWV); and **b** β-stiffness of common carotid artery changes (expressed in percent) in the groups treated with empagliflozin 25 mg daily (EMPA), metformin 2000 mg daily (MET) or empagliflozin/metformin (EMPA/MET). **P < 0.01; ***P < 0.001 for comparison with the metformin group

After 12 weeks’ treatment, empagliflozin on top of metformin and empagliflozin significantly improved PWV compared to metformin treatment (by 15.8% and 14.3%, respectively, both P < 0.01) (Fig. 2a). In the placebo group, PWV values remained unchanged during the course of the study. When compared to placebo, empagliflozin or empagliflozin/metformin significantly improved PWV (up to 5.1- and 5.7-fold, respectively, both P < 0.01).

Carotid β-stiffness was significantly decreased with empagliflozin on top of metformin by 36.6%, the decrease was significantly higher compared to metformin (P < 0.05; Fig. 2b). In the placebo group, β-stiffness values did not change during the course of the study. When compared to placebo, empagliflozin or empagliflozin/metformin significantly improved β-stiffness (up to 3.5- and 4.2-fold, both P < 0.01, respectively).

## Discussion

The potential effect of empagliflozin on top of metformin treatment on arterial function in T1DM patients was extensively studied in the present study. A new insight into the mechanism of the cardiovascular protective effects of empagliflozin was revealed. Empagliflozin, metformin and empagliflozin/metformin improved endothelial function significantly and to a comparable level. Arterial stiffness was maximally decreased in the group treated with the empagliflozin/metformin, the difference was significant compared to metformin group. Direct comparison with metformin revealed similar efficacy in improvement of endothelial function and superior efficacy in reducing arterial stiffness. These results allow two conclusions: (i) empagliflozin and metformin have similar effects on endothelial function and divergent effects on arterial stiffness parameters, and (ii) empagliflozin seems to have a unique capacity (that is independent of endothelial function improvement) to improve arterial stiffness.

This study explored the effects of empagliflozin alone and its effects on top of metformin on structural and functional arterial properties. Metformin, as an optimal comparator, was chosen due to its previously proven beneficial effects on arterial function and reduction of cardiovascular events [17–19]. T1DM patients were chosen, since they were previously naïve for oral antidiabetic agents, and allowed for direct comparison of the effects of empagliflozin and metformin on arterial function. This would be very difficult to realise independently in T2DM patients due to their prior antidiabetic therapy (mainly including metformin) and potential prior modulation of arterial wall function. Empagliflozin on top of metformin significantly decreased arterial stiffness parameters (PWV and carotid β-stiffness) compared to metformin. In contrast, metformin alone improved endothelial function parameters (FMD and RHI) to a comparable extent as empagliflozin or empagliflozin/metformin treatment. The improvement of arterial stiffness by empagliflozin or empagliflozin on top of metformin, but not by metformin alone, suggests the potential specific capacity of empagliflozin to improve arterial stiffness. Furthermore, the improvement of arterial stiffness seemed to be independent from endothelial function, since similar improvement of endothelial dysfunction obtained by metformin was not followed by an improvement of arterial stiffness.

Empagliflozin/metformin also significantly decreased HbA1c values, proving their synergistic effects on glycaemia control, whereas separate agents did not significantly affect HbA1c.

The translation of these results to clinical practice allows for interesting interpretations. The majority of T2DM patients are treated with metformin and addition of SGLT2 inhibitors is one of therapeutic options. Based on our results, metformin alone significantly improves endothelial function and the addition of empagliflozin would not result in significant benefits. Therefore, the cardioprotective effects of empagliflozin cannot be attributed solely to endothelial function improvement. In contrast, adding empagliflozin to metformin therapy would importantly improve arterial stiffness. The degree of expected decrease of arterial stiffness (14% to 18%) would result in an important predicted reduction of cardiovascular risk in the range of 10–20% [3]. Interestingly, a comparable decrease in cardiovascular risk was obtained in the EMPA-REG trial [9].

Several prior studies examined the effects of SGLT2 inhibitors on arterial characteristics. Empagliflozin 25 mg daily significantly decreased systolic blood pressure and several arterial stiffness parameters after 8 weeks of treatment in young T1DM patients, i.e. augmentation indexes at the radial, carotid and aortic positions, carotid-radial PWV [11]. In another study, similar effects were proven for empagliflozin 25 mg daily in T2DM patients after 6 weeks of treatment [13]. Dapagliflozin decreased blood pressure and also improved microcirculation (decrease of retinal capillary flow) after 6 weeks of treatment in T2DM patients [20]. In our study, empagliflozin had no effect on blood pressure. Most probably, the differences in pressure were too small and even less pronounced in patients with normal values to be detected in such a small sample. Improved endothelial function was also observed in Japanese T2DM patients receiving dapagliflozin 5 mg daily [21]. Moreover, in a pilot study, an improvement in FMD and decrease in aortic PWV was recorded even after a short-term, only 2-day treatment with dapagliflozin 10 mg daily in T2DM patients [12]. The protective effects of metformin on endothelial function improvement were found in several studies [22–24]. On the other hand, its effects on arterial stiffness are conflicting. Some studies observed no beneficial effects [25], while others reported such beneficial effects [26]. However, until now, to the best of our knowledge, no studies compared the effects of SGLT2 inhibitors to metformin on arterial function parameters.

The present study is the first of its kind to explore the effect of empagliflozin on arterial function by extensively studying numerous vascular parameters and using a comparator. We observed an improvement in macrovascular endothelial function, which was also found after acute treatment with dapagliflozin in prior studies [12]. The authors speculated that the effect was the consequence of acute oxidative stress reduction, which might be one of the reasons for its non-acute, prolonged effect, as observed in our study. Additional reasons could also be anti-inflammatory action, leading to enhanced NO bioavailability in the arterial wall. Microvascular reactivity was also significantly improved with empagliflozin treatment and similar results were observed in other study that included T2DM patients. Several prior studies also explored the beneficial effects of empagliflozin and dapagliflozin on arterial stiffness parameters [11–13], but were mainly performed in T2DM patients, while only one study included patients with T1DM. Again, the results of the present study are in line with the previous studies. On the other hand, the results in the metformin group are in line with previous observations, where mostly endothelial function was improved [22–24], while no effect on arterial stiffness was observed [25].

Our study is the first to reveal that empagliflozin, even on top of metformin, seems to have a unique capacity to improve arterial stiffness that is not shared by metformin and seems not to be related to improvement of endothelial function. Consequently, it could be deduced that the improvement of arterial stiffness by empagliflozin is not associated to nitric oxide, inflammation and oxidative stress, which are all well-known factors involved in endothelial function improvement [27]. Taken all facts together, it might be speculated that there are receptor/signalling pathway(s) behind improvement of arterial stiffness that are activated directly by empagliflozin. Indeed, some data points that SGLT2 receptors are present on vascular smooth muscle cells (VSMC) [28]. However, the clinical importance of these receptors is unknown. Overall, it seems possible that empagliflozin is a unique, specific activator of arterial stiffness reduction, achieving its effects possibly via SGLT2 receptors on VSMC. Such effects would certainly lead to improved cardiovascular protection as observed in clinical studies [3]. Furthermore, empagliflozin (and, most likely, also other SGLT2 inhibitors) can find a place in treatment of numerous pathological conditions, where increased arterial stiffness is at least part of their pathological processes. The observed beneficial effects of empagliflozin in our cohort of diabetes patients are promising, particularly in terms of prevention of diabetic cardiovascular complications. It is important to emphasise that the measured functional and structural arterial wall changes appear prior to overt cardiovascular disease. Treating such subclinical, or clinically silent disease, might lead to efficacious cardiovascular prevention and reduction in cardiovascular mortality. Similarly, empagliflozin would be also effective in patients with already present cardiovascular diseases. Nevertheless, additional studies on larger populations of diabetes mellitus patients are needed to further explore these effects as well as their underlying mechanisms. Despite its significant results, one of the limitations of the present study is the small number of patients included. That said, it was designed as a pilot study.

## Conclusions

Empagliflozin on top of meformin treatment significantly improved arterial stiffness compared to metformin in T1DM patients. Endothelial function was similarly improved in all treatment groups. Arterial stiffness improvement by empagliflozin was substantial and in ranges that could be of clinical relevance. The effect was independent from endothelial function improvement, suggesting the specific, unique capacity of empagliflozin to improve arterial stiffness. That could support its cardioprotective effects and that of other SGLT2 inhibitors observed in large clinical studies.

## Abbreviations

BMI: body mass index
BP: blood pressure
cfPWV: carotid-femoral pulse wave velocity
FMD: brachial artery flow-mediated dilation
HbA1c: glycated haemoglobin
PWV: pulse wave velocity
RHI: reactive hyperaemia index
SGLT2: sodium glucose co-transporter type 2
T1DM: type 1 diabetes mellitus
T2DM: type 2 diabetes mellitus
